# Development and validation of a quality of life and treatment satisfaction measure in canine osteoarthritis

**DOI:** 10.3389/fvets.2024.1377019

**Published:** 2024-05-03

**Authors:** Edwina Gildea, Emma Scales-Theobald, Jill Thompson, Alasdair Cook, Katie Forde, George Skingley, Sophie Lawrie, Nicola Williamson, Charlotte Panter

**Affiliations:** ^1^Zoetis International, Dublin, Ireland; ^2^School of Veterinary Medicine, University of Surrey, Surrey, United Kingdom; ^3^Adelphi Values Patient-Centered Outcomes, Cheshire, United Kingdom

**Keywords:** canine, chronic pain, osteoarthritis, quality of life, owner, treatment satisfaction

## Abstract

**Introduction:**

Canine osteoarthritis (OA) causes pain and mobility impairment. This can reduce dog quality of life (QoL), owner QoL and owners’ satisfaction with, and adherence to, treatments. No existing canine OA-specific instrument assesses all three impacts. This study aimed to develop and psychometrically evaluate an owner-completed canine OA-specific measure of dog QoL, owner QoL and owner treatment satisfaction; the “Canine OA Quality of Life and Treatment Satisfaction Questionnaire” (CaOA-QoL-TS).

**Methods:**

The CaOA-QoL-TS was developed using a conceptual model derived from a meta-synthesis of published literature followed by cognitive interviews with ten owners of dogs with OA, to evaluate content validity.

**Results:**

Based on interview findings, ten items were reworded, four removed, and two added; resulting in 26 items that all owners understood and considered relevant. The recall period and response options were well understood and appropriate to almost all owners. To evaluate its psychometric properties, the CaOA-QoL-TS (draft 26-item version) was administered, across six timepoints in a phase 4 field study, to owners of OA treated dogs, recruited from veterinary practices (*N* = 93). Inter-item correlations suggested items clustered into three distinct domains: Dog QoL, Owner QoL and Treatment Satisfaction, as hypothesized. Confirmatory factor analysis supported deletion of two items and calculation of the three domain scores, with acceptable model fit. The resulting 24-item CaOA-QoL-TS instrument demonstrated strong internal consistency and good to excellent test–retest reliability. Convergent validity was supported by moderate to strong correlations with concurrent measures. Known groups validity was supported by statistically significant differences between groups categorized by owner global impression of QoL. Ability to detect change was demonstrated through statistically significant improvements over time in Owner and Dog QoL, with larger within-group effect sizes reported for the mean of ‘improved’ dogs compared to the mean of ‘stable’ dogs. Only a small sample of dogs worsened throughout the study. Anchor-based analyses supported-0.9 and-1.0-point within-group responder definitions for dog and owner QoL domains, respectively.

**Discussion:**

Findings support the content validity of the CaOA-QoL-TS in canine OA. The 24-item CaOA-QoL-TS is a reliable and valid instrument to measure owner and canine QoL and TS and is sensitive to improvements following OA treatment.

## 1 Introduction

Osteoarthritis (OA) is a slowly progressive and incurable disease, characterized by degeneration and remodeling of the synovial joints, leading to impaired mobility and chronic pain (1, 2). OA is the most common joint disease diagnosed in veterinary medicine (3, 4). A recent study reported nearly two-fifths of dogs (37.3%) presenting for routine preventative care or evaluation of lameness/stiffness were diagnosed with OA following the use of a pet owner OA screening checklist (5). Early signs and symptoms of OA are often undetected by dog owners as the signs of chronic pain in dogs are often subtle and responses to pain highly individual. When signs of OA are detected, they can often be mistaken as frailty related to old age, leading to late-stage diagnosis (6). It is recognized that radiography is considered part of a diagnostic process however it is also widely acknowledged that radiographic changes do not always corelate with the degree of pain associated with OA nor the impact on mobility. In a real-world setting veterinarians often rely upon their clinical expertise including the dogs medical history, discussion with the owner (s), physical exams and behavioral changes when diagnosis OA (7).

While OA-specific clinical metrology instruments (8–12) have been designed to only measure pain intensity and functional limitations caused by the disease, pain associated with canine OA has also been reported to negatively affect several aspects of a dog’s quality of life (QoL). In the context of veterinary practice, QoL is used to describe the overall wellbeing (13) of animals and may include various aspects of their life such as their physical health, pain, behavior, and emotional state. QoL impacts associated with canine OA include willingness to be physically active (e.g., reluctance to jump or climb stairs), general activity levels, interactions with humans and other animals (e.g., sociability and play, pain-related aggression), food consumption (e.g., appetite loss) and gait (e.g., stiffness, lameness) (7, 14, 15). VetMetrica (VM), an online generic health-related quality of life (HRQL) instrument (16–18) which measures emotional as well as physical wellbeing and accordingly measures the affective (how it makes you feel) component of the OA pain experience, has been validated in dogs with OA (19). However, to date there are no canine OA-specific QoL instruments reported in the literature. Such instruments are valuable in veterinary practice to monitor disease progression and help guide treatment decisions and in clinical trials as outcome measures.

Canine OA has also been reported to have a negative impact on owner QoL. In the context of owners of companion animals, QoL is used to describe the overall wellbeing of the owner in relation to their pet and may include owner perceptions regarding the impact their pet’s health has on their own physical health, emotional/psychological state and social relationships (20). Reported impacts on owner QoL, as a result of canine OA, include stress associated with their dog’s diagnosis, concerns about how to best optimize disease management and, impacts on activities of daily living (ADL) such as walks with their dog (e.g., walking distance, speed, locations) (21, 22). Nonetheless, there are no known canine OA specific instruments that assess the impact of canine OA on the owner’s QoL.

Pain management in canine OA typically involves multiple treatment options including both pharmacological and non-pharmacological therapies. Owner burden and owner treatment satisfaction (i.e., the extent to which individuals perceive a treatment to fulfill health needs) (23) have been identified as key factors when choosing current and/or future treatment options, including the option to euthanize. For example, owners of dogs with severe OA-related pain and functional impairment (24, 25) are less likely to euthanize their dog if they are happy with their dog’s treatment and the treatment is perceived to reduce caregiver burden. For animals with chronic or terminal illnesses, such as OA, adherence to treatment can also be influenced by owner treatment satisfaction and owner burden, including dosing regimen, consultation time, method of administration, palatability of oral medications (26), and perceptions of impaired QoL for both the pet and owner. Assessing owner treatment satisfaction and QoL for both the pet and owner may provide valuable insights into the likelihood of canine OA treatment adherence, support discussions regarding canine OA treatment options in veterinary practice, and, if used in clinical studies, may provide supportive evidence of treatment benefit.

Taking into consideration the far-reaching consequences of canine OA, a composite canine OA-specific instrument that assesses dog QoL, owner QoL and owner treatment satisfaction could prove useful given the influence of these effects on treatment selection and treatment adherence in canine OA. Furthermore, an instrument such as this might help facilitate wider communication and dissemination of findings in the field of pain management in canine OA and help support veterinarian and owner discussions regarding treatment options and outcomes.

The current study builds on research conducted by Zoetis and the University of Surrey, which developed a 29-item draft canine OA-specific instrument, namely the “Canine OA Quality of Life and Treatment Satisfaction Questionnaire” (CaOA-QoL-TS), to assess dog QoL, owner QoL and owner treatment satisfaction. As part of its development, a meta-synthesis of published literature was conducted including an initial literature review resulting in the development of a conceptual model of the QoL impact concepts in dogs with OA (27). Four domains were identified: mobility, behavior, mood, and physical appearance. The impacts of canine OA on owner QoL and the factors influencing owner satisfaction with treatment were also identified. Following this, a second top-up review was conducted to further explore owner QoL and treatment satisfaction which identified substantial impacts on owner emotional wellbeing, physical functioning, ADL, and social functioning (7, 21, 22), as well as the role of treatment efficacy and mode of administration in treatment satisfaction (28–30). The methods used to develop the instrument were aligned with the United States (US) Food and Drug Administration patient-focused drug development guidance series for the development and validation of clinical outcome assessments (COAs), which has subsequently been applied to the development of QoL assessments in companion animals (31, 32).

The current study aimed to evaluate whether the CaOA-QoL-TS can be considered fit-for-purpose in canine OA, by generating qualitative evidence to assess content validity and quantitative evidence to assess the measurement properties of the instrument including the instrument structure, reliability, construct validity, item level analyses, dimensionality analyses, ability to detect changes over time, and meaningful change thresholds.

## 2 Materials and methods

### 2.1 Study design

**Figure 1:**
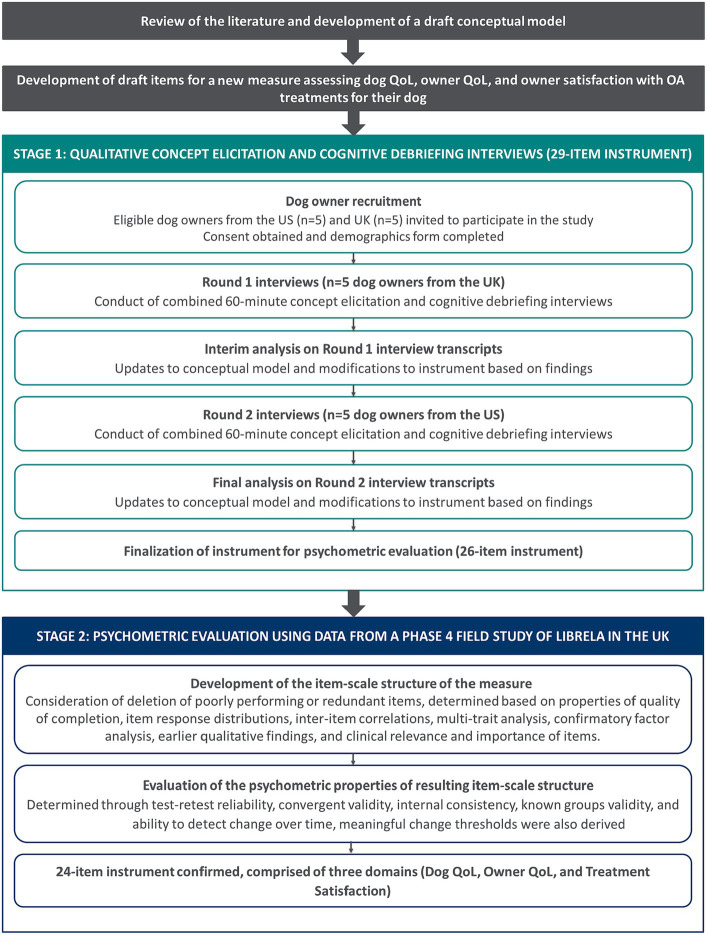
Overview of study design.

Study activities were completed in two stages, an overview of study is detailed in Figure 1 below:

- Stage 1 comprised ten 60-min combined qualitative concept elicitation (CE) and cognitive debriefing (CD) telephone interviews with owners of dogs with a veterinarian-diagnosis of OA in the US (*n* = 5) and United Kingdom (UK; *n* = 5) to explore owner’s experiences of canine OA; this informed revisions to the conceptual model and evaluated the conceptual comprehensiveness of the instrument. Interviews were conducted in two rounds to allow for modifications and testing of the updated instrument between rounds.
- Stage 2 comprised psychometric evaluation of the CaOA-QoL-TS using data from a phase 4 field study of Librela in the UK; this informed item reduction, development of scoring, and evaluation of measurement properties, including reliability, construct validity, ability to detect change over time, and assessment of meaningful change thresholds. Note, the results of the Librela field study are not presented in this manuscript.

### 2.2 COAs included in the study

The following instruments were administered in the study:

- The initial draft 29-item CaOA-QoL-TS instrument – included in Stage 1: round 1 interviews.
- Updated draft 30-item CaOA-QoL-TS instrument – included in Stage 1: round 2 interviews.
- The updated draft 26-item CaOA-QoL-TS instrument – included in Stage 2 psychometric evaluation.
- Four global impression items – included in Stage 1: round 2 interviews and Stage 2 psychometric evaluation.
- The VM Dog instrument – included in Stage 2 psychometric evaluation, intended for convergent validity analyses.

#### 2.2.1 The CaOA-QoL-TS instrument

The owner-completed CaOA-QoL-TS instrument was designed to assess three hypothesized domains: Dog QoL, Owner QoL, and Treatment Satisfaction. The instrument was refined based on round 1 interview findings and was finalized following completion of all stages of the current study; the original draft instrument comprised 29-items (consisting of Dog QoL [17 items], Owner QoL [eight items], Treatment Satisfaction [four items]).

The Dog QoL and Owner QoL domains had a recall period of seven-days; the Treatment Satisfaction domain asked owners to think about their dogs “most recently prescribed treatment for his/her arthritis.” The recall periods were considered appropriate by the instrument developers as they reflected the stability of OA symptom presentation (i.e., limited/no day-to-day variance) and aimed to standardize reporting, while minimizing measurement or recall error and respondent burden (33). Items were rated using a five-point verbal rating scale from ‘Not at all’ to ‘A great deal’ or ‘Not at all’ to ‘Very much’ with some items including a ‘Not applicable’ option for owners who had not observed their dog perform an activity in the past 7 days (i.e., jump up and/or jump down). A five-point verbal rating scale was used to ensure adjacent response options were distinct and reflect true differences in observable signs of canine OA. Item 9 (‘My dog has wanted to go on walks or play’), Item 11 (‘My dog has appeared happy’), and Item 13 (‘My dog has been sleeping well’) of the draft CaOA-QoL-TS instrument were reverse scored. Reverse scoring involves attributing numeric values in the opposite direction on the response scale to ensure conceptual alignment between items and consistent interpretation of the item scores that form the domain score. The draft CaOA-QoL-TS instrument included in the CD interviews was completed by owners in pen and paper format; the CaOA-QoL-TS instrument was migrated to an electronic COA (eCOA) for inclusion in the phase 4 field study.

#### 2.2.2 Global impression items

Four single-item owner-completed global impression items were developed, in line with regulatory guidance (33–35), as anchor measures to support the analyses in the psychometric evaluation study, as described below. The four global impression items included in CD interviews (round 2) were completed by owners in pen and paper format; these items were migrated to an eCOA for inclusion the phase 4 field study.

- Global Impression of Dog Quality of Life (OGID-QoL) – this item asked owners, *“Thinking about your dog’s arthritis, please choose the answer that best describes your dog’s quality of life over the past 7 days”* with a five-point verbal rating scale (‘Poor,’ ‘Fair,’ ‘Good,’ ‘Very good,’ ‘Excellent’).
- Global Impression of Owner Quality of Life (OGIO-QoL) *–* this item asked owners, *“Thinking about your dog’s arthritis, please choose the answer that best describes your quality of life over the past 7 days”* with a five-point verbal rating scale (‘Poor,’ ‘Fair,’ ‘Good,’ ‘Very good,’ ‘Excellent’).
- Global Impression of Dog Quality of Life Change (OGID-C) – this item asked owners, *“Thinking about your dog’s arthritis, please choose the answer that best describes the overall change in your dog’s quality of life, since your dog started his/her most recently prescribed arthritis treatment”* with a five-point verbal rating scale (‘Much better,’ ‘Better,’ ‘No change,’ ‘Worse,’ ‘Much worse’).
- Global Impression of Owner Quality of Life Change (OGIO-C) – this item asked owners, *“Thinking about your dog’s arthritis, please choose the answer that best describes the overall change in your quality of life, since your dog started his/her most recently prescribed arthritis treatment”* with a five-point verbal rating scale (‘Much better,’ ‘Better,’ ‘No change,’ ‘Worse,’ ‘Much worse’).

#### 2.2.3 The VM dog instrument

The owner-completed 22-item VM Dog instrument is an existing instrument aimed to assess chronic pain and its impact on QoL in dogs (17–19, 36, 37). While not specific to canine OA, it has been validated as a measure of QoL related impacts associated with chronic pain in dogs with OA (19) and was therefore included to evaluate convergent validity of the CaOA-QoL-TS instrument. The VM Dog instrument was completed by owners as an eCOA in the phase 4 field study.

### 2.3 Stage 1: qualitative CE and CD interviews

#### 2.3.1 Sample and recruitment

Participants were identified and recruited through the authors’ network of veterinarians in the US and UK. Veterinarians reviewed their databases for dogs who met the study eligibility criteria and contacted their owners to invite them to participate. To be eligible, owners had to be at least 18 years of age at the time of recruitment and the owner of a dog diagnosed with OA, in at least one limb, by a veterinarian. Veterinarian-diagnosis of OA was confirmed through clinical expertise including detailed history of the dog, conversations with owner (s) and physical examination. A diagnosis through radiography was not required as the methods above reflect diagnosis of OA in real-world settings. Further, all dogs included were on prescriptions that require a veterinarian-confirmed diagnosis of OA. Owner and dog demographic characteristics were collected to characterize the sample. Information collected about the owner included: age, gender, education level, the type of area they lived in, the length of time they had owned a dog, and length of time they had owned a dog with OA. Information collected about the dog included: age, sex, breed, date of OA diagnosis, current severity of OA and current health status. Owners were also required to complete the Canine Brief Pain Inventory item assessing the dogs QoL in the past 7 days. Participants were compensated for their time. All participants provided written informed consent prior to commencing any study-related activities.

The target sample size was informed by the principles of conceptual saturation (38), while ensuring study feasibility and a diverse sample by the dog’s age, sex, breed, and OA severity. Although samples of 12 can typically achieve conceptual saturation (i.e., the point at which no new relevant concepts are likely to emerge with further interviews) (39), in this instance, as the sample was expected to be homogenous, the sample of 10, combined with the findings of the systematic literature review and resulting conceptual model, was judged to be sufficient to achieve saturation.

#### 2.3.2 Interview procedure

The CE section of the interviews used broad, open-ended questions to facilitate spontaneous elicitation of concepts regarding the impact of canine OA on dog and owner QoL. Owners were asked to describe how canine OA affects their dog’s behavior and whether they believe any signs or behaviors are indicators of good or poor QoL in dogs, as well as how their dog’s OA affects their own QoL. Focused questions were used if concepts of interest identified in the literature reviews had not emerged or been fully explored. Owners were also asked questions about their dog’s current arthritis treatment, to explore treatment satisfaction.

For the CD section, owners in round 1 and round 2 interviews were asked to complete the draft CaOA-QoL-TS instrument using a ‘think aloud’ approach and to share their thoughts as they read each instruction/item and selected their response. Owners in round 2 were also asked to complete the four global impression items on paper using the same approach. Owners were then asked detailed questions about their interpretation and understanding of the instructions/item wording, relevance of concepts, and appropriateness of the response options and recall period. CD of the instrument was completed following CE to minimize bias in the topics participants chose to discuss.

#### 2.3.3 Qualitative analysis

All interviews were audio-recorded, via Microsoft Teams, and transcribed verbatim by an accredited transcription vendor. The CE section of the interview transcripts were subject to thematic analysis using Atlas. Ti software, version 8 (40). Participant quotes were assigned corresponding concept codes in accordance with a coding scheme agreed amongst the research team. Throughout the data analysis process, the study team met on a regular basis to resolve any discrepancies through discussion and consensus-building. The code list was updated iteratively and organically throughout the analysis, and previously coded transcripts were revisited and reviewed to identify any instances where the new codes may apply. Codes were applied both deductively (based on prior knowledge) and inductively (as emerging from the data). An assessment of conceptual saturation was conducted for CE data to ensure data collection was exhaustive. For this, transcripts were chronologically grouped into two sets of three and one set of four. Spontaneously reported QoL and treatment satisfaction concepts emerging from each set were then iteratively compared. Saturation was considered met if no new concept-relevant information emerged in the final set of interviews (38, 39). The CD section of the transcripts were analyzed using dichotomous codes that were assigned to each instruction, item, response option, and recall period to indicate whether it was understood and relevant.

### 2.4 Stage 2: phase 4 field study

A multi-center, uncontrolled, prospective, longitudinal, phase 4 field study of Librela was conducted in the UK. A sample size of 120 owners of dogs aged ≥12 months, with a veterinarian-confirmed diagnosis of OA, was targeted to produce at least 90 evaluable cases (accounting for a hypothesized 25% attrition rate). The study comprised six timepoints: Day 0 (Baseline), Day 14 (2 weeks after first dose), Day 28 (second dose), Day 56 (third dose), Day 63 (1 week following third dose) and Day 70 (2 weeks following third dose). The six COA measures: CaOA-QoL-TS, VM Dog, OGID-QoL, OGIO-QoL, OGID-C, and OGIO-C; were completed at all six timepoints on an electronic device. All participants provided written informed consent prior to commencing any study-related activities. Psychometric analyses using data collected from this study were conducted to evaluate the measurement properties of the CaOA-QoL-TS.

#### 2.4.1 Psychometric analysis

An analysis plan was written prior to the conduct of any analyses. Analyses were conducted in two phases, as detailed in Supplementary Table 1. Phase A aimed to develop the item-scale structure of the draft CaOA-QoL-TS that would be taken forward to Phase B, including consideration of deletion of poorly performing or redundant items. This was determined based on the properties of quality of completion, item response distributions, inter-item correlations, multi-trait analysis, confirmatory factor analysis (CFA), earlier qualitative findings, and the clinical relevance and importance of items. Phase B analyses (test–retest reliability, convergent validity, internal consistency, known groups validity, and ability to detect change over time) evaluated the psychometric properties of the resulting item-scale structure. All analyses used SAS version 9.4 (41), apart from CFA which was performed using Mplus run in R and calculation of coefficient omega using R version 4.2.2 (42).

## 3 Results

### 3.1 Qualitative interview results

#### 3.1.1 Sample characteristics

Ten owners (female: *n* = 9, male: *n* = 1; mean age [range]: 49.8 [32–75] years) of dogs, with a veterinarian-confirmed diagnosis of OA, (female: *n* = 6, male: *n* = 4; mean age [range]: 12.7 [9–16] years), based in the US (*n* = 5) and UK (*n* = 5) participated in an interview. Most lived in a rural area (*n* = 7, 70.0%) and had a college degree or equivalent (*n* = 8, 80.0%). On average, participants had owned a dog for 12.7 years and had owned a dog with OA for 9.5 years. Dogs varied in breed and included a range of small and large dogs, see Supplementary Table 2.

#### 3.1.2 CE results

##### 3.1.2.1 Dog and owner QoL

Owners reported a total of 31 QoL impacts that they had observed in their dog, which were categorized into seven core themes: mobility, sleep, toileting, vocalizations, energy, temperament/mood, and physical appearance. The most frequently reported impacts related to mobility, including difficulty jumping up/down (*n* = 9) and difficulty getting up/lying down (*n* = 8).

In terms of their own QoL, owners reported a total of 27 QoL impacts related to their dog’s OA. Impacts on emotional wellbeing (*n* = 9; e.g., worry, guilt) were most frequently reported, followed by physical functioning (*n* = 8; e.g., need to lift or carry dog) and ADL (*n* = 7; e.g., need to plan life around treatment). Six owners described the need to modify their physical environment to accommodate their dog’s OA (e.g., installing stairs/ramp to aid in climbing) and five described how their social functioning (e.g., pre-planning to ensure suitable environment/transport) and sleep (e.g., night-time awakenings) had been impacted by their dog’s OA. The financial cost of managing their dog’s OA, including prescribed treatments and supplements, physical therapy, and environmental adaptations, was also noted by four owners; one owner described impacts on their work.

##### 3.1.2.2 Treatment satisfaction

Satisfaction with their dog’s OA treatment was discussed by nine owners. Owners explained their experience with their dogs current OA treatment and indicated which aspects of treatment that would impact their level of satisfaction. Treatment satisfaction concepts were grouped into five themes: mode of administration, treatment efficacy, frequency of administration, ease of fitting treatment into daily life, and availability of treatment options. Owners indicated that they would be satisfied with a treatment that is readily available and effective at reducing signs of canine OA, easy to administer, fit into their daily life, and requires less frequent administration (relative to other available treatment options).

##### 3.1.2.3 Updated conceptual model

**Figure 2:**
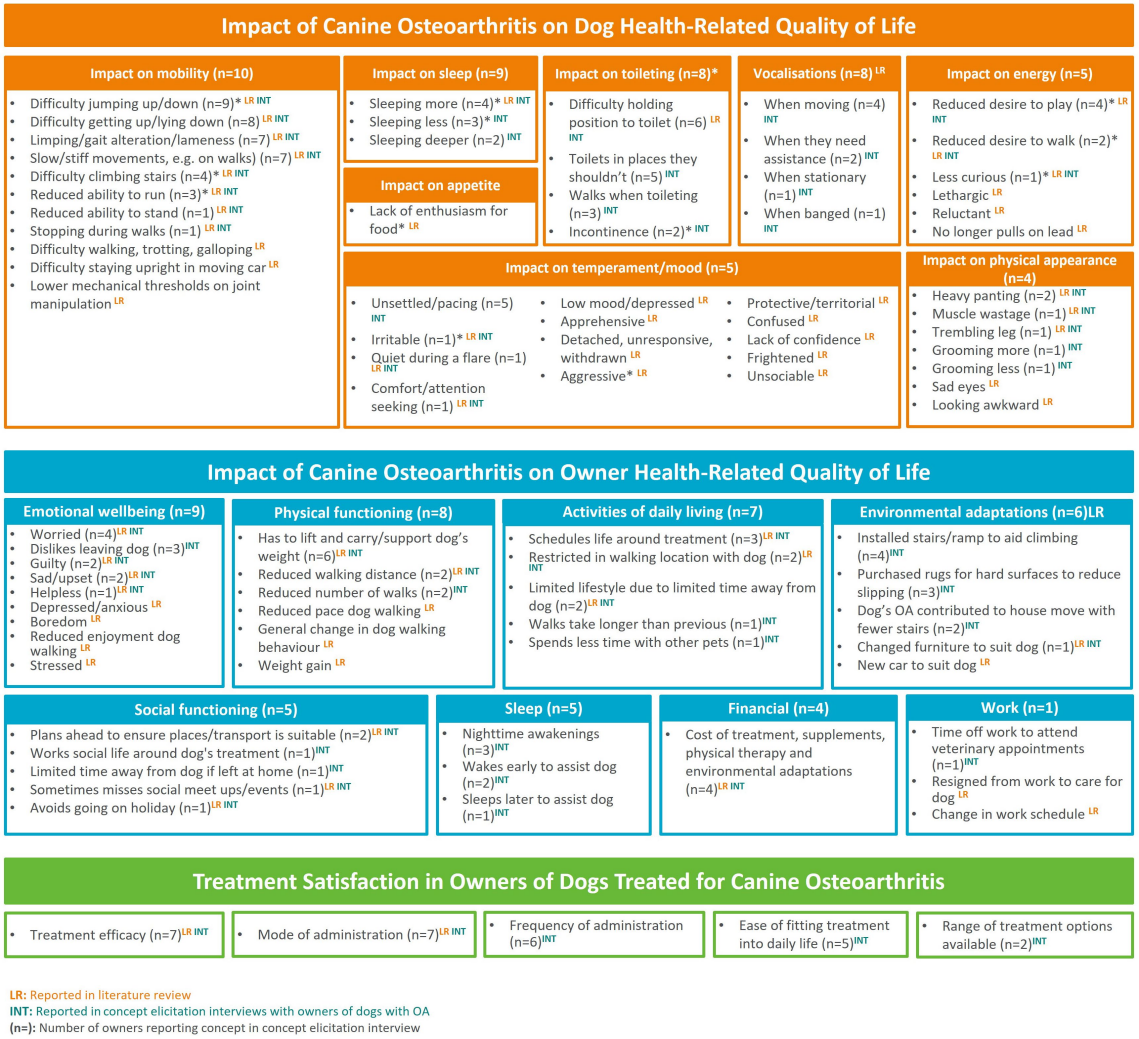
Updated conceptual model of the impact of canine OA on dog and owner QoL and treatment satisfaction.

Based on the CE findings, the conceptual model, developed following earlier literature review findings, was updated to include data from the interviews completed in the current study (Figure 2). Saturation analysis highlighted that no new concepts were identified in the last set of interviews.

#### 3.1.3 CD results

##### 3.1.3.1 Round 1

In round one, five dog owners completed and debriefed the draft 29-item CaOA-QoL-TS. Most items (28/29) were understood by ≥80% of participants and items included in the Dog and Owner QoL domains (22/25) were considered relevant to ≥60% of participants. Items assessing dogs’ ‘difficulty climbing down steps or stairs,’ and owners’ ‘physical activity’ and ‘enjoyment in going for walks with dog’ were relevant to ≤40% of participants. Relevance was not assessed for the Treatment Satisfaction domain.

Most instructions were understood however, two participants had difficulty understanding whether the recall period used in the Treatment Satisfaction domain referred to treatments prescribed in the past week or if it referred to treatments prescribed prior to the past week. Participants demonstrated understanding of the response options (*n* = 5/5) and considered them appropriate (*n* = 4/4; one participant did not explicitly report if they considered the response options appropriate, or not). One participant suggested including a ‘Not applicable’ option for the Dog QoL domain to account for any behaviors they do not allow their dog to do.

##### 3.1.3.2 Updates following round 1

Based on the round 1 findings, several modifications were made to the CaOA-QoL-TS instrument. In addition to updates made to the item wording (summarized in Supplementary Figure 1), updates to the item recall period and response scale included:

- The removal of the recall period (‘in the past 7 days’) from the instructions and items in the Treatment Satisfaction domain, to improve comprehension.
- Reduction in the number of response options in the Treatment Satisfaction domain to four options, to reflect changes in the instruction wording.
- The addition of a ‘Not applicable’ response option to select items of the Dog QoL domain for behaviors that were not observed or allowed by owners (e.g., ‘I do not allow, or have not seen, my dog to climb up or down steps or stairs’).

##### 3.1.3.3 Round 2

In round 2, five dog owners completed and debriefed the 30-item CaOA-QoL-TS and four global impression items. Findings indicated that all 30 CaOA-QoL-TS items were understood by all participants. Most items of the Dog and Owner QoL domains (20/25) were also considered relevant to ≥60% of participants. Items assessing dogs’ ‘difficulty climbing up and/or down steps’ and ‘wanting to go on walks or play’ and, owners’ ‘social interactions’, ‘enjoyment in going for walks with dog’, and ‘difficulty letting others look after dog’ were relevant to ≤40% of participants. For Item 5 (‘difficulty climbing up and/or down steps or stairs’) two participants noted that they did not allow their dog to do this activity. As above, relevance was not assessed for items in the Treatment Satisfaction domain.

All participants understood the instructions as intended, suggesting that modifications to the Treatment Satisfaction domain improved participant comprehension. All participants demonstrated understanding of the response options and considered them appropriate. All participants understood the global impression items as intended and without difficulty. All five participants who were asked also demonstrated an understanding of the global impression item response options and considered them appropriate.

##### 3.1.3.4 Updates following round 2

Based on the round 2 findings, further modifications were made to the CaOA-QoL-TS instrument. In addition to the removal of items (summarized in Supplementary Figure 1), minor updates to the ‘not applicable’ response option were made to the Dog QoL domain, for select items, to ensure that following migration from pen and paper to an eCOA, all items fit on a screen.

**Figure 3:**
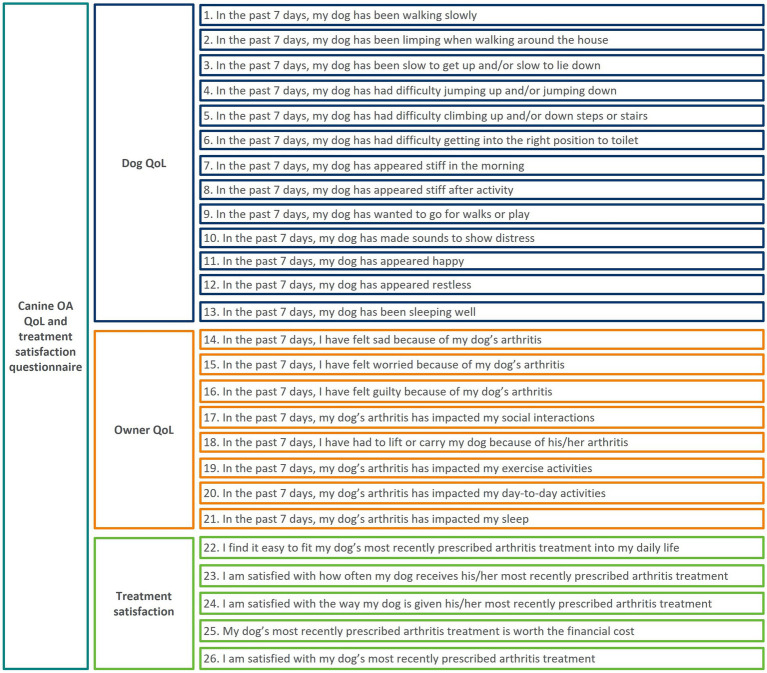
Updated conceptual framework of CaOA-QoL-TS questionnaire, following conduct of qualitative interviews.

This resulted in a 26-item instrument taken forward for the phase 4 field study and psychometric evaluation (Figure 3). No modifications were made to the global impression items.

### 3.2 Psychometric evaluation results

#### 3.2.1 Sample characteristics

A total of 93 dog-owners completed the CaOA-QoL-TS at all timepoints in the phase 4 field study. Neutered female dogs made up half (49.5%) of the sample included in the analyses followed by neutered males (20.4%), males (18.3%) and females (11.8%). Dogs had a mean age of 10.3 years (range: 0–22.0 years) (see Supplementary Table 2). Owner demographics were not collected as part of the validation study as they were not pertinent to the study objectives. Dogs varied in breed and included a range of small and large dogs with a total over 26 different breeds included, in addition to mixed-breeds and ‘other’ (see Supplementary Table 2).

#### 3.2.2 Phase A: development of the item-scale structure of the CaOA-QoL-TS

##### 3.2.2.1 Quality of completion

Form level quality of completion was high (73.1%), narrowly missing the 25% attrition rate hypothesized in the analysis plan. Due to electronic administration of the instrument prohibiting respondents to skip items, item level quality of completion was not assessed.

##### 3.2.2.2 Item response distributions

Item-level response distributions, as assessed at Baseline, demonstrated a good spread of responses across the response scale for most items, except for Item 23 (‘satisfaction with frequency of treatment’), Item 25 (‘financial cost of treatment’), and Item 26 (‘overall satisfaction with treatment’).

Floor and ceiling effects were considered present in an item when at least 50% of respondents scored the worst possible score (floor) or best possible score (ceiling) on the response scale. No floor effects were observed for any items at any timepoint. A ceiling effect was identified at Baseline for Item 6 (‘difficulty getting in position to toilet’; 51%), Item 10 (‘made sounds to show distress’; 53%) and Item 21 (‘impacted my sleep’; 66%). In the Treatment Satisfaction domain, between 55.0 and 82.0% of respondents reported the highest score possible on each item (i.e., very much). Ceiling effects were also observed at later timepoints, as expected.

##### 3.2.2.3 Inter-item correlations

Inter-item correlations were calculated using data from Day 28 to provide an initial exploration of dimensionality. In general, items correlated more strongly with items within their hypothesized domains (Table 1). Inter-item correlations between eight item pairs were notably strong (Polychoric correlation >0.80) and the items were flagged for further consideration. A very strong correlation (*r* = 0.90) was observed between items 14 (‘feeling sad’) and 15 (‘feeling worried’), indicating potential redundancy. Weak correlations (<0.40) were observed for Item 9 (‘wanted to go on walks/play’) and Item 13 (‘sleeping well’) with most other items in their hypothesized domain. Item 18 (‘lift/carry’) of the Owner QoL domain was the only item to have >50% shared variance with an item outside its domain (Item 4 ‘difficulty jumping up/down’ of the Dog QoL domain; *r* = 0.77).

**Table 1:** Inter–item correlation matrix.

Item	1	2	3	4	5	6	7	8	9	10	11	12	13	14	15	16	17	18	19	20	21	22	23	24	25	26
1. Walking slow	1.00	–	–	–	–	–	–	–	–	–	–	–	–	–	–	–	–	–	–	–	–	–	–	–	–	–
2. Limping	0.51	1.00	–	–	–	–	–	–	–	–	–	–	–	–	–	–	–	–	–	–	–	–	–	–	–	–
3. Slow to get up/lie down	0.59	0.65	1.00	–	–	–	–	–	–	–	–	–	–	–	–	–	–	–	–	–	–	–	–	–	–	–
4. Difficulty jumping	0.51	0.59	0.76	1.00	–	–	–	–	–	–	–	–	–	–	–	–	–	–	–	–	–	–	–	–	–	–
5. Difficulty climbing	0.64	0.51	0.72	0.79	1.00	–	–	–	–	–	–	–	–	–	–	–	–	–	–	–	–	–	–	–	–	–
6. Position to toilet	0.34	0.49	0.62	0.51	0.50	1.00	–	–	–	–	–	–	–	–	–	–	–	–	–	–	–	–	–	–	–	–
7. Stiff in morning	0.48	0.54	0.82	0.68	0.57	0.60	1.00	–	–	–	–	–	–	–	–	–	–	–	–	–	–	–	–	–	–	–
8. Stiff after activity	0.55	0.69	0.73	0.64	0.47	0.60	0.74	1.00	–	–	–	–	–	–	–	–	–	–	–	–	–	–	–	–	–	–
9. Wanted to walk/play	0.40	0.31	0.22	0.45	0.38	0.33	0.33	0.34	1.00	–	–	–	–	–	–	–	–	–	–	–	–	–	–	–	–	–
10. Sounds of distress	0.22	0.38	0.52	0.48	0.39	0.51	0.53	0.62	0.37	1.00	–	–	–	–	–	–	–	–	–	–	–	–	–	–	–	–
11. Happy	0.22	0.38	0.37	0.49	0.40	0.47	0.38	0.54	0.68	0.67	1.00	–	–	–	–	–	–	–	–	–	–	–	–	–	–	–
12. Restless	0.27	0.23	0.41	0.38	0.34	0.41	0.51	0.56	0.37	0.65	0.45	1.00	–	–	–	–	–	–	–	–	–	–	–	–	–	–
13. Sleeping well	0.00	−1.00	−0.45	0.14	0.03	0.05	0.12	0.31	0.24	0.40	0.35	0.46	1.00	–	–	–	–	–	–	–	–	–	–	–	–	–
14. Sad	0.53	0.53	0.62	0.59	0.54	0.40	0.48	0.57	0.38	0.48	0.40	0.24	0.04	1.00	–	–	–	–	–	–	–	–	–	–	–	–
15. Worried	0.43	0.50	0.54	0.57	0.51	0.41	0.41	0.58	0.34	0.45	0.49	0.29	0.05	0.90	1.00	–	–	–	–	–	–	–	–	–	–	–
16. Guilty	0.37	0.48	0.44	0.38	0.35	0.40	0.40	0.52	0.23	0.49	0.40	0.34	0.08	0.70	0.81	1.00	–	–	–	–	–	–	–	–	–	–
17. Social interactions	0.59	0.61	0.63	0.58	0.48	0.36	0.44	0.56	0.38	0.60	0.44	0.34	0.13	0.76	0.78	0.68	1.00	–	–	–	–	–	–	–	–	–
18. Lift/carry	0.36	0.29	0.60	0.77	0.65	0.46	0.50	0.42	0.40	0.48	0.48	0.27	0.16	0.57	0.54	0.37	0.44	1.00	–	–	–	–	–	–	–	–
19. Exercise	0.50	0.50	0.49	0.41	0.36	0.43	0.34	0.55	0.33	0.40	0.31	0.39	0.22	0.64	0.70	0.66	0.85	0.30	1.00	–	–	–	–	–	–	–
20. Day–to–day activities	0.57	0.58	0.65	0.60	0.51	0.46	0.53	0.68	0.42	0.45	0.43	0.44	0.17	0.74	0.76	0.61	0.86	0.47	0.89	1.00	–	–	–	–	–	–
21. Sleep	0.40	0.30	0.40	0.45	0.45	0.36	0.34	0.54	0.46	0.66	0.67	0.60	0.55	0.51	0.55	0.45	0.58	0.43	0.62	0.60	1.00	–	–	–	–	–
22. Easy to fit treatment in life	−0.26	−0.24	0.03	−0.13	−0.02	0.06	−0.10	−0.14	−0.14	−0.19	−0.23	−0.24	−0.31	0.05	0.13	−0.05	0.07	0.02	0.13	0.26	−0.04	1.00	–	–	–	–
23. Frequency of treatment	−0.40	−0.36	−0.22	−0.32	−0.22	0.04	−0.28	−0.32	−0.28	−0.39	−0.47	−0.36	−0.35	−0.23	−0.14	−0.17	−0.35	−0.07	−0.19	−0.17	−0.39	0.73	1.00	–	–	–
24. Way treatment received	−0.37	−0.26	−0.23	−0.23	−0.15	0.05	−0.17	−0.10	−0.15	−0.40	−0.26	−0.08	−0.22	−0.23	−0.04	−0.14	−0.21	−0.11	−0.18	−0.06	−0.26	0.72	0.84	1.00	–	–
25. Financial cost of treatment	−0.29	−0.19	−0.19	−0.13	−0.13	0.07	−0.22	−0.23	−0.24	−0.46	−0.31	−0.38	−0.37	−0.24	−0.11	−0.19	−0.22	−0.08	−0.25	−0.12	−0.36	0.68	0.77	0.73	1.00	–
26. Overall satisfaction	−0.46	−0.38	−0.44	−0.27	−0.35	−0.00	−0.32	−0.38	−0.28	−0.44	−0.41	−0.32	−0.33	−0.39	−0.21	−0.20	−0.35	−0.20	−0.28	−0.37	−0.55	0.60	0.76	0.72	0.83	1.00

##### 3.2.2.4 Dimensionality of the CaOA-QoL-TS

The appropriateness of grouping the items into three hypothesized multi-item scores (comprising 26 items) was tested using multi-trait analysis and CFA. Multi-trait analysis was conducted using data from Day 28 and involved correlating individual items with each domain score; items were expected to have stronger correlations with their hypothesized domain score. Most items correlated at least moderately (>0.40) with their hypothesized domain except Item 13 (‘sleeping well’; *r* = 0.19), indicating it could be a candidate for removal. All items correlated stronger with their hypothesized domain, except Items 18 (‘lift/carry’; Owner QoL = 0.46, Dog QoL = 0.61) and 21 (‘sleep impacted’; Owner QoL = 0.54, Dog QoL = 0.60), highlighting the need for further inspection.

CFA was conducted using data from Day 28 to assess the fit of the hypothesized model to the data. Standardized factor loadings of items on their hypothesized domains all exceeded 0.40 except for Item 13 (‘sleeping well’), which loaded on the Dog QoL domain at 0.28. The Dog and Owner QoL domains were strongly related (0.77); however, both correlated negatively with the Treatment Satisfaction domain (−0.45 and − 0.27, respectively) as scoring is interpreted in the opposite direction for this response scale. Fit statistics for this model were not considered acceptable (comparative fit index = 0.943; root mean square error of approximatio*n* = 0.084; standardized root mean square residual = 0.109). Modification indices indicated that adding Item 18 (‘lift/carry’) to the Dog QoL domain would have the greatest impact on model fit.

##### 3.2.2.5 Item reduction

Overall, these data suggest that Item 13 (‘sleeping well’) does not fit with the other items in the Dog QoL domain. The wording of Item 18 asks whether the owner needs to lift or carry their dog due to his/her arthritis, rather than the impact of this activity on the owner (the item stem for the other items in this domain), which could explain why it appears as an item to measure dog rather than owner QoL. Item response distributions cross-tabulated by dog weight categories revealed differences in patterns of responses based on dog weight. Considering this, along with item wording, general item performance and clinical relevance of the item, the decision was made to remove Item 18 from the CaOA-QoL-TS (Supplementary Figure 1).

Further, based on these findings and importantly considering the findings from the previous qualitative research, Item 13 was also removed from the CaOA-QoL-TS. Although sleep impacts were reported by most owners in the CE interviews (*n* = 9/10), the specific impacts were broad, ranging from their dog sleeping less to sleeping more or deeper. Nearly half of the participants debriefed on the item also reported that the item was not relevant (i.e., their dogs sleep was not affected by OA; *n* = 2/5) (Supplementary Figure 1).

**Figure 4:**
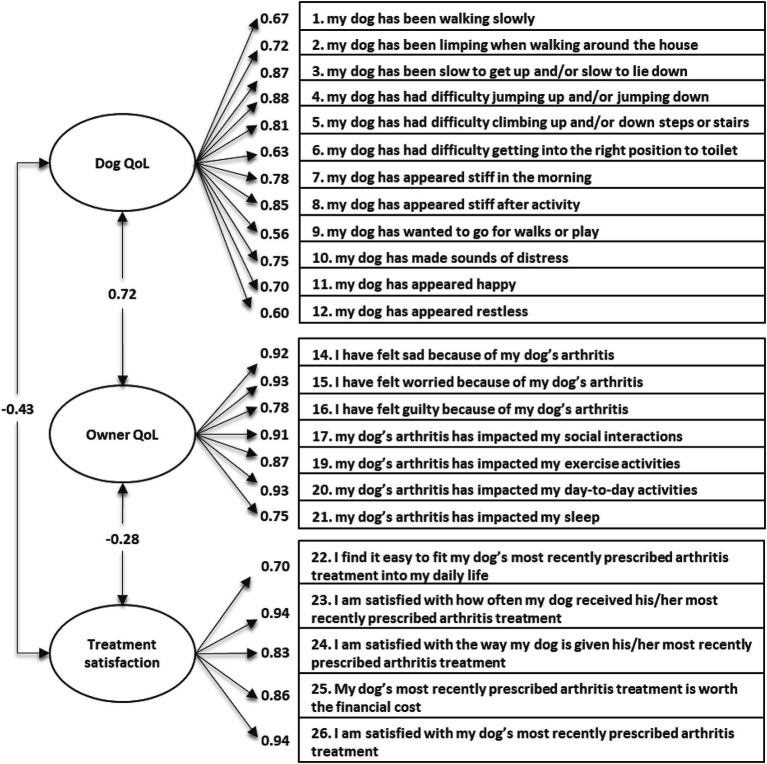
Confirmatory factor analysis factor loadings of the final CaOA-QoL-TS instrument at Day 28 (*n* = 93).

Repeating the CFA without Items 13 (‘sleeping well’) and 18 (‘lift/carry’) saw an improvement in fit statistics (comparative fit index = 0.966; root mean square error of approximatio*n* = 0.071; standardized root mean square residual = 0.099, Figure 4), suggesting that this revised structure was acceptable. All items correlated well (>0.40; Table 2) with their domain score, supporting item-convergent validity through multi-trait analysis. Most items correlated higher with their respective domains; the exception being Item 21 (‘sleep impacted’; QoL domain), which still had a slightly stronger correlation with the Dog QoL domain (*r* = 0.58) compared with the Owner QoL domain (*r* = 0.53). However, both correlations were strong (>0.50). Following item removal results supported the structure of the CaOA-QoL-TS as three separate domain scores.

**Table 2:** Multi-trait analysis of the items in each hypothesized domain at Day 28 (*n* = 93).

Scale	Item	Polyserial item-scale correlations		
		Dog QoL	Owner QoL	Treatment satisfaction
Dog QoL	1. My dog has been walking slowly	0.564	0.509	−0.375
	2. My dog has been limping when walking around the house	0.633	0.550	−0.255
	3. My dog has been slow to get up and/or slow to lie down	0.793	0.562	−0.216
	4. My dog has had difficulty jumping up and/or jumping down	0.777	0.535	−0.200
	5. My dog has had difficulty climbing up and/or down steps or stairs	0.729	0.485	−0.202
	6. My dog has had difficulty getting into the right position to toilet	0.604	0.423	0.025
	7. My dog has appeared stiff in the morning	0.753	0.444	−0.212
	8. My dog has appeared stiff after activity	0.759	0.591	−0.214
	9. My dog has wanted to go for walks or play^ⴕ^	0.440	0.372	−0.161
	10. My dog has made sounds to show distress	0.590	0.485	−0.332
	11. My dog has appeared happy^ⴕ^	0.576	0.447	−0.276
	12. My dog has appeared restless	0.498	0.351	−0.235
Owner QoL	14. I have felt sad because of my dog’s arthritis	0.655	0.775	−0.221
	15. I have felt worried because of my dog’s arthritis	0.635	0.814	−0.067
	16. I have felt guilty because of my dog’s arthritis	0.536	0.672	−0.143
	17. My dog’s arthritis has impacted my social interactions	0.653	0.829	−0.200
	19. My dog’s arthritis has impacted my exercise activities	0.526	0.776	−0.144
	20. My dog’s arthritis has impacted my day-to-day activities	0.655	0.780	−0.100
	21. My dog’s arthritis has impacted my sleep	0.582	0.525	−0.315
Treatment Satisfaction	22. I find it easy to fit my dog’s most recently prescribed arthritis treatment into my daily life	−0.117	0.109	0.592
	23. I am satisfied with how often my dog received his/her most recently prescribed arthritis treatment	−0.358	−0.231	0.725
	24. I am satisfied with the way my dog is given his/her most recently prescribed arthritis treatment	−0.222	−0.160	0.694
	25. My dog’s most recently prescribed arthritis treatment is worth the financial cost	−0.271	−0.192	0.760
	26. I am satisfied with my dog’s most recently prescribed arthritis treatment	−0.431	−0.332	0.694

^ⴕ^ Symbol means that the item was reverse scored.

#### 3.2.3 Phase B: evaluation of the psychometric properties of the resulting item-scale structure

##### 3.2.3.1 Internal consistency

Internal reliability of the Dog QoL, Owner QoL, and Treatment Satisfaction domains was examined using data from Day 28. Cronbach’s alpha values were well above the priori threshold of ≥0.70 (Cronbach’s alpha = 0.90, 0.91, and 0.86, respectively), providing evidence for internally consistent (homogenous) scales. Omega coefficients values were also calculated at Day 28 using omega (total) and demonstrated good reliability >0.70 (Dog QoL = 0.90, Owner QoL = 0.91, Treatment Satisfactio*n* = 0.87) for all three domain scores of the CaOA-QoL-TS.

##### 3.2.3.2 Test–retest reliability

Stable dogs were defined as those whose QoL was rated by their owners as ‘stable or unchanged’ between Days 56 and 63 on the OGID-QoL; 53 dogs met this criterion. Similarly, stable owners were defined as those whose QoL was rated as “stable or unchanged” between Days 56 and 63 on the OGIO-QoL; 63 owners met this criterion. For the Treatment Satisfaction domain, it was assumed scores would remain stable throughout the study as treatment did not change; therefore, all dog-owner pairs were included in the analysis (*n* = 93). Test–retest reliability was good (ICC > 0.75) for the Owner QoL and Treatment Satisfaction domain scores and excellent (ICC > 0.90) for the Dog QoL domain score (Table 3).

**Table 3:** Test–retest reliability.

Scale	Test–retest reliability					
	Reliability (ICC)	95% confidence intervals		Day 56 Mean (SD)	Day 63 Mean (SD)	Pearson correlation coefficient
		Lower	Upper			
Dog QoL	0.91	0.85	0.95	0.92 (0.83)	0.88 (0.74)	0.915
Owner QoL	0.81	0.70	0.88	0.70 (0.92)	0.62 (0.74)	0.828
Treatment satisfaction	0.88	0.83	0.92	3.55 (0.72)	3.57 (0.69)	0.883

##### 3.2.3.3 Convergent validity

To evaluate convergent validity, correlations of the Dog QoL and Owner QoL domains with the VM Dog domain scores, OGID-QoL, and OGIO-QoL were evaluated using data collected at Day 28 (Table 4). Stronger correlations were expected between measures of similar constructs. As predicted, results demonstrated evidence of convergence between the Dog QoL domain and all domains of the VM Dog (range − 0.48 to −0.68) and the OGID-QoL (−0.53). However, the OGIO-QoL correlated more with the Dog QoL domain (−0.56) than the Owner QoL domain (−0.52), which may be a functioning of the relatively high correlation between these two domains in the factor analysis. For the Owner QoL domain, all correlations were moderate to strong (−0.31 to −0.52), exceeding the hypothesized level for measures expected to correlate weakly (<0.30) with this domain. Despite this, correlations with the Dog QoL domain were stronger for those measures more related to the construct of interest and therefore were not of concern.

**Table 4:** Convergent validity Pearson correlations at Day 28 (*n* = 93).

Instrument/item score	Dog QoL domain	Owner QoL domain
VM Dog-Active/Comfortable	−0.68	−0.50
VM Dog-Calm/Relaxed	−0.48	−0.31
VM Dog-Energetic/Enthusiastic	−0.62	−0.45
VM Dog-Happy/Content	−0.51	−0.32
OGID-QoL	−0.53	−0.41
OGIO-QoL	−0.56	−0.52

##### 3.2.3.4 Known groups analyses

The Dog QoL domain score was compared among groups who differed in overall QoL as measured by the OGID-QoL. There was a pattern of significantly higher mean scores (indicating worse QoL) for dogs who also scored higher (worse) on the OGID-QoL on Day 28 (*p* < 0.001), with the expected monotonic increases across the groups. Large effect sizes between the ‘poor or fair’ and ‘good’ groups (−1.31) and the ‘poor or fair’ and ‘very good or excellent’ groups (−1.97) further provide evidence for known groups validity (Table 5).

**Table 5:** Known-group analysis of the dog QoL and owner QoL domains (*n* = 93).

Domain	Comparison group	*n*	Mean (SD)	Median	Between group effect size	*p*-value
Dog QoL	Poor or fair	7	1.88 (0.77)	1.92	Ref.	<0.001
	Good	30	1.12 (0.53)	1.08	−1.31	
	Very good or excellent	56	0.75 (0.55)	0.75	−1.97	
Owner QoL	Poor or fair	4	1.64 (1.24)	1.29	Ref.	<0.001
	Good	31	1.22 (0.84)	1.00	−0.48	
	Very good or excellent	58	0.48 (0.67)	0.29	−1.63	

A pattern of higher scores (indicating worse QoL) was also reported by owners who reported worse scores on the OGIO-QoL, with statistically significant differences between group means for the Owner QoL domain score (*p* < 0.001). Effect sizes were small between the ‘poor or fair’ and ‘good’ groups (−0.48) and large between the ‘poor or fair’ and ‘very good or excellent’ groups (−1.63), indicating varying levels of difference in mean scores between groups (Table 5).

##### 3.2.3.5 Ability to detect change

Within-group effect sizes and between-groups one-way ANOVA F-test were calculated to evaluate the magnitude and significance in change scores between each group (improved/worsened versus stable) for the Dog and Owner QoL domains (Table 6). As the Treatment Satisfaction domain had no anchors, a descriptive summary of change for each timepoint from Baseline was produced to descriptively explore the ability to detect change.

**Table 6:** Ability to detect change.

Anchor/domain	Comparison group	*n*	Mean change score (SD)	Median change score	Min, Max	Within group effect size	Between groups *p*-value
Dog QoL domain between Baseline and Day 56							
OGID-QoL	Improved: ≥1 grade improvement	61	−1.26 (0.74)	−1.42	−3.00, 1.33	−1.76	<0.001
	Stable: no change	23	−0.41 (0.50)	−0.33	−1.17, 0.33	−0.59	
	Worsened: ≥1 grade worsening	9	0.12 (0.86)	0.00	−0.92, 1.75	0.23	
OGID-C	Improved: “Better” or “Much better”	77	−1.09 (0.73)	−1.17	−3.00, 0.33	−1.53	<0.001
	Stable: no change	13	−0.20 (0.70)	−0.08	−1.42, 1.33	−0.27	
	Worsened: “Worse” or “Much worse”	3	0.57 (1.50)	1.08	−1.12, 1.75	0.43	
Owner QoL domain between Baseline and Day 56							
OGIO-QoL	Improved: ≥1 grade improvement	58	−1.31 (0.86)	−1.14	−3.86, 0.57	−1.44	<0.001
	Stable: no change	21	−0.59 (0.92)	−0.71	−1.86, 2.29	−0.70	
	Worsened: ≥1 grade worsening	14	−0.07 (1.16)	−0.14	−2.57, 2.57	−0.08	
OGIO-C	Improved: “Better” or “Much better”	66	−1.21 (0.89)	−1.14	−3.86, 0.29	−1.28	<0.003
	Stable: no change	26	−0.47 (0.96)	−0.71	−2.43, 2.29	−0.60	
	Worsened: “Worse” or “Much worse”	1	2.57 (N.A.)	2.57	2.57, 2.57	N.A.	

For the Dog QoL domain score, change scores between groups were statistically significant for both the OGID-QoL and OGID-C (*p* < 0.001 each), from Baseline to Day 56. Effect sizes were larger for the improved group compared to the stable group, providing strong evidence of the ability to detect improvement in Dog QoL. Although the sample size for the worsened group was small, findings support that worse Dog QoL is associated with higher scores on the Dog QoL domain, as would be expected.

Similarly, for the Owner QoL domain score, change scores between groups were statistically significant for both the OGIO-QoL (*p* < 0.001) and OGIO-C (*p* = 0.003), from Baseline to Day 56, providing evidence of the ability to detect change. Due to the small sample size for the worsened group, no effect size could be produced for the OGIO-C, and a small effect size in the wrong direction for worsening (i.e., negative change score) was observed for the OGIO-QoL, providing no clear evidence of the ability to detect change in worsening of Owner QoL. However, for both the OGIO-QoL and OGIO-C effect sizes were larger for the improved group compared to the stable group, providing evidence of the ability to detect improvement in the Owner QoL domain.

For the Treatment Satisfaction domain score, there were minimal changes from Baseline in mean scores observed across Days 14 to 70 (0.06–0.10); therefore, it was not possible to detect changes over time in Treatment Satisfaction. Given the known clinical effectiveness of the trial treatment (43–45), stability in Treatment Satisfaction score was expected and not a concern.

##### 3.2.3.6 Meaningful change

###### 3.2.3.6.1 Anchor-based methods

Change correlations in CaOA-QoL-TS domain scores from Baseline to Day 56 showed that the OGID-QoL (*r* = 0.74) and OGID-C (*r* = 0.58) were appropriate for use as anchors for the Dog QoL domain, and the OGIO-QoL (*r* = 0.53) and OGIO-C (*r* = 0.54) were appropriate for use as anchors for the Owner QoL domain, correlating >0.30.

The between-group minimal important difference (MID) estimate for the Dog QoL domain was −0.59 for the OGID-QoL and −0.56 for the OGID-C. For the Owner QoL domain the MID estimate was −0.55 for the OGIO-QoL and −0.42 for the OGIO-C. This suggested a between-group MID of around −0.50 would be applicable for both the Dog and Owner QoL domains.

Calculation of within-group mean change scores for the ‘minimally improved’ group provided a range of estimates (−0.76 to −1.00, 95% confidence interval (CI) range: −0.53 to −1.26) for the Dog QoL domain, and a similar range (−0.89 to −1.13, 95% CI range: −0.64 to −1.34) for the Owner QoL domain. Triangulation of these ‘minimally improved’ group estimates provided a single responder definition of −0.9 for the Dog QoL domain and of −1.0 for the Owner QoL domain (rounded to one decimal place for ease of interpretation). Anchor-based evaluation of meaningful change for the Treatment Satisfaction domain was not explored as no suitable anchors were available; change in Treatment Satisfaction was considered less relevant than the raw score itself given only overall satisfaction with treatment was expected to change with the administration of the same treatment over time.

The empirical cumulative distribution function (eCDF) plots are displayed in the Supplementary materials (Supplementary Figures 2, 3) and show that each responder definition classifies a low proportion of respondents who are worsened or stable as improved, while classifying a high proportion (>60%) of minimally improved or much improved respondents as improved.

###### 3.2.3.6.2 Distribution-based methods

For the Dog QoL domain ½SD = 0.37, SEM = 0.29; Owner QoL domain ½SD = 0.45, SEM = 0.35. For the Dog and Owner QoL domains these values were smaller than the correlation weighted average anchor-based responder definitions indicating these definitions exceed measurement error.

## 4 Discussion

The CaOA-QoL-TS instrument was developed, following best practice methods for COA development and regulatory guidance (33–35, 46), to address the paucity of canine OA-specific measures. In addition, the instrument was designed to not only assess dog QoL, but also owner QoL and owner satisfaction with OA treatment. The intention was to offer flexibility in the assessment of these domains by modifying and validating an instrument that can be administered either in its entirety or each domain independently depending on the users’ objectives. This was supported by the confirmation of the three-factor structure and reliability and validity evidence for each independent domain score.

The CaOA-QOL-TS includes QoL impacts and aspects of canine OA treatment that are most important and relevant to owners of dogs with OA, as confirmed by literature reviews and by owners during qualitative interviews (13, 27). Findings relating to the assessment of dog QoL also align with pre-existing instruments that commonly assess activity level and the desire for interaction as indicators of QoL. The qualitative interviews also confirmed that items included in the CaOA-QoL-TS assessing these concepts are relevant and well understood by owners of dogs with OA.

The present study provides evidence that the CaOA-QOL-TS instrument has strong psychometric properties that are key to accurately and reliably assess both dog and owner QoL and owner satisfaction with canine OA treatment over time. All domain scores demonstrated good or excellent internal consistency and test–retest reliability. Notably, known groups analyses provided evidence that the instrument can discriminate among OA dogs of differing levels of owner-reported positive health states and moderate to strong correlations between the dog and owner QoL scores with established measures supported the convergent validity of the instrument. Additionally, anchor-based meaningful change analyses indicated within-group responder definitions for the Dog QoL (−0.9) and Owner QoL (−1.0) domain scores.

While the results from the research are positive, it is important to acknowledge the limitations and highlight its strengths. The instrument’s content validity was assessed via qualitative interviews using a small sample of dog owners (*n* = 10) who were predominately female. However, despite the small sample size and gender skew, the qualitative study sample were varied across demographics hypothesized to influence QoL and treatment satisfaction, including age, educational level, work status, location (rural vs. urban environment in the UK/US), and length of dog ownership (both of OA and non-OA dogs). Importantly the adequacy of the qualitative sample size was determined based on the theory of conceptual saturation whereby no new concepts were emerging from the previous interviews thereby indicating the key concepts relevant to owners of dogs with OA had been reported (47). The demographic and clinical characteristics of the owners during the psychometric evaluation were not collected as they were not deemed relevant to the study objectives. Identical to the qualitative study, owners were recruited directly by their veterinarian therefore the sample is reflective of the owners of dogs with OA in general veterinarian practice and real-world settings.

Regarding the recruitment and inclusion of dogs in the study, while dogs were required to have a veterinarian diagnosis of OA, no formal radiographic imaging was used to clinically confirm this diagnosis. Prior research suggests that there is a lack of consensus amongst veterinarians, pet owners, veterinary physical rehabilitation practitioners and researchers, with regards to how a definitive diagnosis of canine OA is obtained (48). While radiography, and similar imaging techniques, may be perceived as a conclusive method to confirm OA diagnosis, there are known limitations, including its validity. Radiography lacks sensitivity in the early stages of OA detection and radiographic evaluation and clinical disease do not necessarily correlate (49, 50). Attitudes to radiography, amongst veterinary surgeons, has also been found to vary both within and between practices. Very few veterinary surgeons, who took part in practice-based focus groups, routinely offered radiography at their practice; most relied on the dog’s medical history, clinical examination and trial treatment when diagnosing OA (7). In addition, radiography was not perceived to provide additional diagnostic information; instead, veterinary clinical expertise and judgment is typically relied upon when diagnosing canine OA in veterinary practice. As such, the diagnosis of canine OA in this study is reflective of the approach to diagnosing canine OA in real world veterinarian practice which was the setting for the current research and the intended context of use for the CaOA-QoL-TS instrument. Therefore, the absence of verifying a diagnosis of OA via imaging techniques is not thought to impact the integrity or application of the research.

In addition, a range of dog sizes and breeds [both recognized breeds and mixed-breed dogs (i.e., dogs that do not belong to an officially recognized breed)] were included across both stages of the study. Labrador retrievers made up a relatively large percentage of the psychometric sample (20%, see Supplementary Table 2) however this reflects the increased risk of developing OA within this specific dog breed (51). The sample of dogs were relatively old in comparison to the average dog life expectancy (52) and the majority were neutered, however again this is expected given OA is more frequently diagnosed in older and neutered dogs (51).

Owner and canine QoL was not defined in the global items (OGID-QoL, OGIO-QoL, OGID-C, OGIO-C) however, participants’ understanding of these items was assessed. While it is acknowledged that not providing a definition of QoL may have resulted in participants’ interpretating this term in different ways, the findings demonstrated that participants who were asked about their understanding of the term described it as intended by the authors. As such, the addition of a definition of QoL within each global item was deemed unnecessary. Furthermore, despite high completion rates of the CaOA-QoL-TS instrument during the psychometric evaluation study, there was a marginally higher attrition rate than hypothesized. It should be acknowledged that data collection occurred during the COVID-19 pandemic which impacted owners’ ability to attend veterinary practice for routine treatment monitoring appointments.

Finally, it should be noted that the draft CaOA-QoL-TS instrument was debriefed with owners of dogs with OA in pen and paper format, however the electronic version was completed in the phase 4 field study. While it is recognized best practice to perform equivalence testing, additional testing was not deemed necessary given only minor changes were made between migration of the CaOA-QoL-TS to an eCOA; (49) any measurement error or bias, created by changing the mode of assessment, was perceived to be too small to affect the assessment of the concept of interest (33, 53, 54). The response options utilized by the CaOA-QoL-TS, global items and VM Dog instrument are also more compatible with migration to an eCOA, as opposed to a VAS, therefore testing was also not deemed necessary. During the phase 4 field study owners did not report any difficulties using the electronic version of the instruments, thus supporting its usability in eCOA format (55, 56).

To the authors’ knowledge, the CaOA-QoL-TS is the only validated instrument for use in canine OA that allows for the comprehensive assessment and monitoring of Dog QoL, Owner QoL, and Treatment Satisfaction within one single instrument, with the ability to use the domains independently depending on the required assessment needs. Given the signs of canine OA are commonly mistaken by pet owners as signs of frailty in aging that results in a late diagnosis and delayed treatment; the Dog QoL domain can be used to assess canine OA-specific signs and symptoms. Domains can be administered in veterinary practice to monitor OA, to improve management of the condition, help inform treatment decisions, and measure response to medical interventions. The Dog QoL domain also offers a standardized assessment of treatment efficacy when testing OA interventions with dogs in OA and can be used to support endpoints in clinical research. Given the documented impact of chronic health conditions of pets on the wellbeing of pet owners (21, 22, 28–30), the Owner QoL domain plays a critical role in providing a more holistic view of OA impact and can be used to facilitate communications regarding the benefit of canine OA treatments from the owner’s perspective. The Treatment Satisfaction domain can be used to assess the owner’s satisfaction with pharmacological canine OA treatments, the output of which can be used by stakeholders to communicate the value of treatment interventions. In addition, in acknowledgement that treatment satisfaction negatively correlates with treatment adherence (30), the Treatment Satisfaction domain can be used in veterinary practice to guide treatment decisions, and also help inform the development of canine OA treatments to optimize both efficacy and adherence.

## 5 Conclusion

This study provides evidence that the CaOA-QoL-TS instrument has strong reliability and content and construct validity to assess Dog QoL, Owner QoL and Treatment Satisfaction in canine OA. Depending on the objective of the assessment, the instrument can either be administered in its entirety or each domain can be used as an independent tool to inform veterinary decision making, support stakeholder communications in the field of pain management, or to support study endpoints in future clinical research.

## Data availability statement

The original contributions presented in the study are included in the article/Supplementary material, further inquiries can be directed to the corresponding author.

## Ethics statement

Ethical approval was not required for the studies involving animals in accordance with the local legislation and institutional requirements because of the methods and nature of the research conducted. All research was conducted in accordance with the World Medical Association’s Declaration of Helsinki and General Data Protection Regulation. All participants provided their written informed consent prior to participating in any study-related activities. Written informed consent was obtained from the owners for the participation of their animals in this study.

## Author contributions

EG: Writing – review & editing. ES-T: Writing – review & editing. JT: Writing – review & editing. AC: Writing – review & editing. KF: Conceptualization, Methodology, Investigation, Formal analysis, Writing – original draft, Writing – review & editing. GS: Conceptualization, Methodology, Investigation, Formal analysis, Writing – original draft, Writing – review & editing. SL: Methodology, Investigation, Formal analysis, Writing – original draft, Writing – review & editing. NW: Methodology, Investigation, Formal analysis, Writing – original draft, Writing – review & editing. CP: Conceptualization, Methodology, Investigation, Formal analysis, Writing – review & editing.
