# Supplementary material for: Development and validation of a quality of life and treatment satisfaction measure in canine osteoarthritis
Source: Front Vet Sci. 2024 May 3;11:1377019. doi: 10.3389/fvets.2024.1377019 (PMC11100416; doi:10.3389/fvets.2024.1377019)
Supplement: Supplementary file 1 [file Table_1.docx]

| Supplementary Table 1. Summary of psychometric analyses | |
| --- | --- |
| Analysis | Description |
| Phase A: Item-level and dimensionality analyses | |
| Item response distributions | Item distributions (frequency and percentage of dog owners selecting each response) were assessed for all timepoints and items with a large proportion (>50%) of owners selecting the best (ceiling effect) or worst (floor effect) possible options were considered further. |
| Inter-item correlations | Correlations between items (inter-item) assessed the homogeneity of items per domain and items correlating >0.90 were considered potentially redundant. |
| Multi-trait analysis | Multi-trait analysis examined whether items in the same domain were correlated adequately. Items in the same domain were expected to correlate more highly (>0.40) with items in their own domain, demonstrating item-convergent validity (58). |
| Confirmatory factor analysis | CFA evaluated the hypothesized three domain conceptual framework. Model fit was assessed using comparative fit indices (CFI), root mean square error approximation (RMSEA), and standardized root mean square residual (SRMR); CFI ≥0.95 (59), RMSEA and SRMR <0.10 were considered a good model fit (60). |
| Phase B: Reliability and validity | |
| Internal consistency | Internal consistency, concerned with the homogeneity of items, was assessed using Cronbach’s alpha coefficient (>0.70 for good internal consistency) (61) and Omega coefficients values using omega (total).(62) Internal consistency was assessed using data from Day 28. |
| Test-retest reliability | Test-retest reliability evaluated consistency in scores between Day 56 and Day 63 in a subset of stable dogs/owners, using intra-class correlation coefficients (ICCs; >0.75 for good test-retest reliability) (63, 64). Only owners and dogs for whom owners reported ‘no change’ on the OGIO-C and OGIO-D were defined as ‘stable’ and included in the analysis. It was assumed that scores for Treatment Satisfaction would remain stable; thus, all owner-dog pairs were included in the analysis. |
| Convergent validity | Convergent validity was evaluated using data collected at Day 28, by examining the correlations of scores of the VM dog (all four domains) and anchor measures (OGID-QoL and OGIO-QoL). Scores assessing similar or related concepts were expected to have strong correlations (≥0.50), whereas scores assessing unrelated concepts were expected to show small (<0.30) or negligible correlations. |
| Known groups analysis | Construct validity was also assessed using the known groups method to evaluate differences in scores among owners/dogs who differ on variables hypothesized to influence the construct of interest. Groups were defined by responses to the OGID-QoL or OGIO-QoL (categories: ‘poor/fair’, ‘good’, ‘very good/excellent’). The magnitude of the differences was considered using between-group effect estimates (small change=0.20, moderate change=0.50, large change=0.80) (65). F-test calculated by one-way ANOVAs (comparison of more than two groups) were used to evaluate if differences were statistically significant (*p* ≤0.05). |
| Ability to detect change | Ability to detect change assesses whether a score fluctuates in line with true change in the construct it measures. Between and within-groups mean change scores were compared from Baseline to Day 56 for OGID-QOL and OGIO-QoL (categories: improved=≥1 grade improvement, stable=no change, worsened=≥1 grade worsening) and for OGID-C and OGIO-C (categories: improved= ‘much better/better’, stable=‘no change, worsened=‘worse/much worse’), using one-way ANOVA F-test (between group) and within-group effect sizes (66). |
| Deriving meaningful  change threshold | Meaningful change thresholds characterize how meaning is attributed to observed changes and differences in scores, beyond what is provided for by statistically significant results.  Anchor-based methods were of primary interest with distribution-based methods used as supportive. In anchor-based methods, an easily interpretable and patient-centered external indicator is used to identify dogs/owners who have experienced an improvement on the concept being measured (OA-related QoL). Anchor-based thresholds included within-group responder definitions and between-group minimal important differences (MID) estimated using OGID-QoL, OGIO-QoL, OGID-C, OGIO-C as anchors.  Anchor-based change scores had to correlate >0.30 with the domain change score to be considered relevant for interpretation [49, 51, (67)]. Thresholds were calculated by evaluating within and between group mean change scores. The within-group responder definition estimates were calculated using mean change scores for the ‘Minimally improved’ (i.e., 1-point OGID-QoL improvement) group from Baseline to Day 56. The between-group MID was defined as the difference between the mean change scores from Baseline to Day 56 for the ‘Minimally improved’ and ‘Stable’ groups for each anchor. To determine a single within-group responder definition estimates were triangulated using a correlation weighted average with Fisher’s Z transformation to produce a single value threshold [52, (68)]. Empirical cumulative distribution function (eCDF) plots were produced to evaluate the performance of the responder definitions.  Distribution-based estimates of ½ standard deviation (SD) at Baseline and the standard error of measurement (SEM) at Baseline were calculated to identify the amount of change exceeding measurement error. |
