# Supplementary material for: Development and validation of a quality of life and treatment satisfaction measure in canine osteoarthritis
Source: Front Vet Sci. 2024 May 3;11:1377019. doi: 10.3389/fvets.2024.1377019 (PMC11100416; doi:10.3389/fvets.2024.1377019)
Supplement: Supplementary file 2 [file Table_2.docx]

| Supplementary Table 2. Dog demographic characteristics for each sample | | |
| --- | --- | --- |
| **Sample characteristics** | **Qualitative interviews**  **(*n*=10)** | **Psychometric study**  **(*n*=93)** |
| Age of dog (years)  Mean (SD) | 12.7 (2.3) | 10.3 (3.3) |
| Sex of dog, n (%)  Female  Neutered female  Male  Neutered male | 6 (60.0%)  -^a^  4 (40.0%)  -^a^ | 11 (11.8%)  46 (49.5%)  17 (18.3%)  19 (20.4%) |
| Weight of dog (Kg)  Mean (SD) | -^a^ | 23.7 (11.6) |
| Breed of dog, n (%)  Labrador  Mixed breed  Staffordshire Bull Terrier  Border Collie  Other  Golden Retriever  Cocker Spaniel  Chihuahua  Springer Spaniel  Shih Tzu  Rottweiler  Pug  Poodle  Jack Russell Terrier  Flat Coated Retriever  Border Terrier  German Shepherd  Brittany Spaniel  West Highland Terrier  Belgian Shepherd  Siberian Husky  Schnauzer  Pomeranian  Newfoundland  Maltese Terrier  Labradoodle  Irish Terrier  Greyhound  English Pointer  Clumber Spaniel  Boxer | -  6 (60.0%)  -  -  -  -  -  -  -  -  -  -  -  -  -  -  1 (10.0%)  1 (10.0%)  1 (10.0%)  1 (10.0%)  -  -  -  -  -  -  -  -  -  -  - | 19 (20.4%)  16 (17.2%)  8 (8.6%)  6 (6.5%) 5 (5.4%) 4 (4.3%)  4 (4.3%)  3 (3.2%)  2 (2.2%)  2 (2.2%)  2 (2.2%)  2 (2.2%)  2 (2.2%)  2 (2.2%)  2 (2.2%)  2 (2.2%)  1 (1.1%)  -  -  -  1 (1.1%)  1 (1.1%)  1 (1.1%)  1 (1.1%)  1 (1.1%)  1 (1.1%)  1 (1.1%)  1 (1.1%)  1 (1.1%)  1 (1.1%)  1 (1.1%) |
| Months since OA diagnosis  Mean (range) | 36.3 (1-96) | -^a^ |
| Owner-rated current health status of dog  Good  Very good  Fair  Poor | 6 (60.0%)  2 (20.0%)  1 (10.0%)  1 (10.0%) | -^a^  -^a^  -^a^  -^a^ |
| Owner-rated current OA severity of dog  Mild  Moderate  Severe | 5 (50.0%)  3 (30.0%)  2 (20.0%) | -^a^  -^a^  -^a^ |
| CBPI quality of life in past 7 days  Excellent  Very good  Good  Fair | 3 (30.0%)  3 (30.0%)  2 (20.0%)  2 (20.0%) | -^a^  -^a^  -^a^  -^a^ |
| ^a^ Information not collected | | |
