# Supplementary material for: Development and validation of a quality of life and treatment satisfaction measure in canine osteoarthritis
Source: Front Vet Sci. 2024 May 3;11:1377019. doi: 10.3389/fvets.2024.1377019 (PMC11100416; doi:10.3389/fvets.2024.1377019)
Supplement: Supplementary file 3 [file Image_1.pdf]

| | Development of draft items | Updates made following review of literature and conceptual model | Updates made following round 1 interviews (n=5) | Updates made following round 2 interviews (n=5) | Psychometric validation in phase 4 field study |
|---|---|---|---|---|---|
| **Dog QoL** | **13 draft items** assessing dog HRQoL developed based on preliminary literature review and conceptual model | 3 items **removed** that overlapped with other items or were not supported by the literature (e.g., 'easy for dog to move and exercise').<br>1 item **added** ('heavy panting') following secondary review of the literature and conceptual model.<br>3 items assessing multiple concepts (e.g., 'limping or stiff') **split** into single items to form 6 additional items. | 1 item **added** ('sleeping well') following round 1 interim analysis<br>3 item-pairs judged to assess sufficiently similar concepts based on interview findings **merged** to form 3 single items ('difficulty jumping up/down', 'slow to get up/down', 'difficulty climbing up/down'). | 2 items **removed** ('heavy panting', 'uncomfortable) due to lack of conceptual relevance or inconsistent interpretation. | 1 item **removed** ('sleeping well') based on poor psychometric performance and consideration of qualitative findings. |
| **Owner QoL** | **11 draft items** assessing owner HRQoL developed based on preliminary literature review and conceptual model | 3 items **removed** that overlapped/were better assessed by other items or were unlikely to improve with treatment (e.g., 'worry about the financial costs of caring for dog's OA'). | 2 items **added** ('sleep', 'lift/carry') following round 1 interim analysis | 2 items **removed** ('enjoyed walking dog', 'letting others look after dog') due to lack of conceptual relevance. | 1 item **removed** ('lift/carry') based on poor psychometric performance and consideration of qualitative findings. |
| **Treatment satisfaction** | **10 draft items** assessing owner satisfaction with OA treatment developed based on preliminary literature review and conceptual model | 6 items **removed** that were considered too broad, which could lead to inconsistent interpretation, overlapped with other items, were not relevant, or considered inappropriate (e.g., 'dog is comfortable on current OA treatment') | 1 item **re-added** ('dog's current OA treatment worth the financial cost') following round 1 interim analysis | No changes made | No changes made |
| | **34 draft items** initially developed | **29 items** debriefed in round 1 interviews | **30 items** debriefed in round 2 interviews | **26 item** CaOA-QoL-TS taken forward for psychometric validation | **24 item** CaOA-QoL-TS confirmed |

Supplementary Figure 1. CaOA-QoL-TS development overview
