# Supplementary material for: Development and validation of a quality of life and treatment satisfaction measure in canine osteoarthritis
Source: Front Vet Sci. 2024 May 3;11:1377019. doi: 10.3389/fvets.2024.1377019 (PMC11100416; doi:10.3389/fvets.2024.1377019)
Supplement: Supplementary file 4 [file Image_2.pdf]

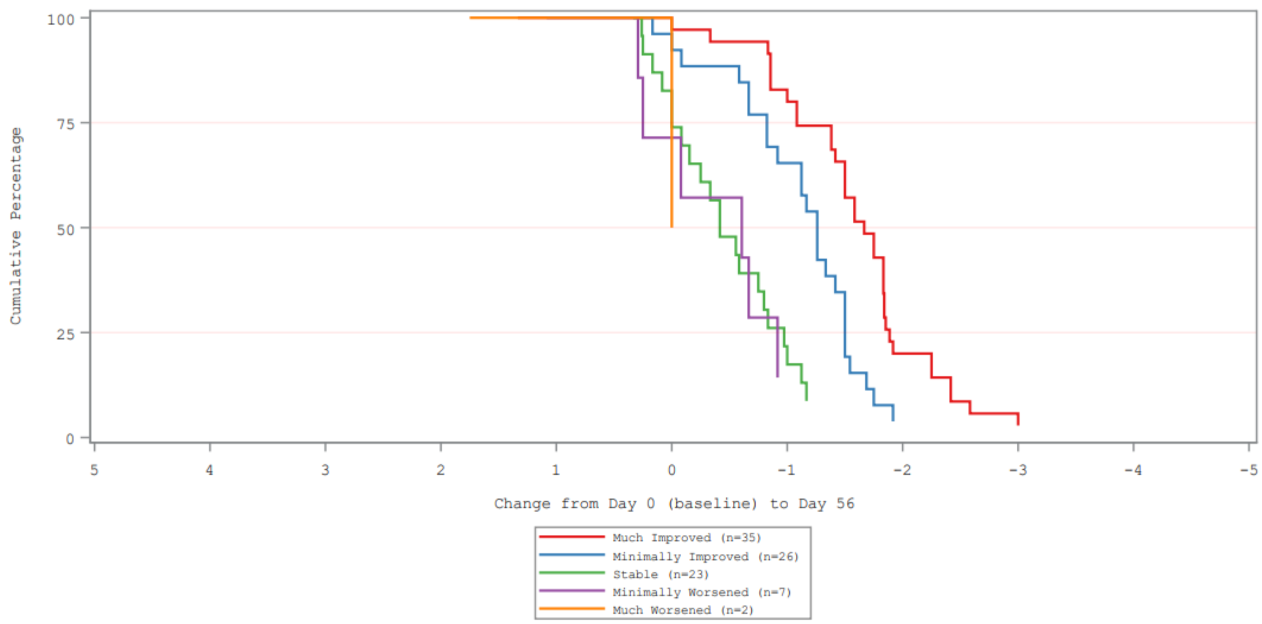

Supplementary Figure 2. Cumulative distribution function of Dog QoL domain score change by OGID-QoL anchor group
